# Analysis of diversity-dependent species evolution using concepts in population genetics

**DOI:** 10.1007/s00285-021-01559-5

**Published:** 2021-02-25

**Authors:** Ingemar Kaj, Sylvain Glémin, Daniah Tahir, Martin Lascoux

**Affiliations:** 1 Department of Mathematics, Uppsala University, Box 480, Uppsala, 751 06 Sweden; 2 CNRS, Université Rennes 1, ECOBIO (écosystèmes, biodiversité, évolution) – UMR 6553, Avenue du général Leclerc, 35042 Rennes, France; 3 Program in Plant Ecology and Evolution, Department of Ecology and Genetics, EBC, Uppsala University, Norbyvägen 18D, Uppsala, 752 36 Sweden

**Keywords:** Wright–Fisher diffusion, Two-type branching, Scaling limit process, Trait fixation probability, Carrying capacity model, 60J70, 92D15

## Abstract

In this work, we consider a two-type species model with trait-dependent speciation, extinction and transition rates under an evolutionary time scale. The scaling approach and the diffusion approximation techniques which are widely used in mathematical population genetics provide modeling tools and conceptual background to assist in the study of species dynamics, and help exploring the analogy between trait-dependent species diversification and the evolution of allele frequencies in the population genetics setting. The analytical framework specified is then applied to models incorporating diversity-dependence, in order to infer effective results from processes in which the net diversification of species depends on the total number of species. In particular, the long term fate of a rare trait may be analyzed under a partly symmetric scenario, using a time-change transform technique.

## Introduction

Models of species richness based on simple birth-death mechanisms with constant speciation and extinction rates suffer from the classical dichotomy of supercritical branching processes; species must either go extinct or grow in number without bound. Such models applied to characterizing divergence of living organisms in terms of number of species in taxas and families, consequently fail to produce what is typically observed. The situation is similar for diversification models of multi-trait species based on multi-type, linear branching process theory. To qualify as a species model, at least one trait should have a strictly positive net growth rate, which again leads to the same supercritical dichotomy as for a single trait. See e.g., Haccou et al. (2005) for a general account of branching processes with emphasis on variation, growth and extinction of populations.

In reality, species numbers derived from fossil data or estimated from analysis of phylogenetic trees often tend to vary over long periods of time in a mode of stationarity or quasi-stationarity within a finite range of realistic values. It is, thus, rather natural to seek to implement in the modeling set-up, some form of population size control or diversity-dependent regulation. While logistic-type population-size dependence is successfully implemented in branching process theory and easily interpretable in term of population dynamics, the nature and causes of diversity-dependent diversification are still debated, cf. Lambert (2006), Rabosky (2013). The diversity-independent models studied in e.g., Maddison et al. (2007) and Tahir et al. (2019), assume that the per-lineage rates of speciation, extinction, and transition of traits are trait-dependent but constant in the sense that they do not depend on the current number of species of a particular trait, or the current total number of species. On the other hand, systems involving diversity-dependence retard supercritical growth by regulating the increase in species numbers through birth and death rates in various ways (Quental and Marshall 2010). In the papers of Parsons and Quince (2007a, 2007b), that consider population-level dynamics, the birth rates are assumed to be decreasing functions of the total number of individuals in the population, thus, imposing a maximal carrying capacity of the system in case the birth rates vanish at some level. In logistic branching models, additional deaths are imposed in proportion to the square of the number of individuals to slow down population growth, Lambert (2005). Similarly, Parsons et al. (2008, 2010) apply population size dependent mortality rates. Fournier and Méléard (2004) consider an ecological system where individuals are characterized by their location, and the mortality rate depends on the local population density. In an environment of say two competing traits, both speciation and extinction rates could be diversity-dependent, and the rates could be allowed to have different sensitivity to competition (Mallet 2012). Further variations have been proposed, such as using time varying, instead of fixed, carrying capacities (Marshall and Quental 2016), and including adaptive radiations by decoupling of the diversity-dependent dynamics of a sub-clade (Etienne and Haegeman 2012). Also proposed are models in which positive net diversification rates are followed by negative net diversification rates to produce ‘waxing and waning’ diversity dynamics (Morlon 2014), and models where the speciation rates are decreasing functions of time, such that the magnitude of the decline in speciation increases as time approaches the environment’s carrying capacity (Rabosky and Lovette 2008). Typical examples are discussed in e.g., Rabosky (2013) and Etienne et al. (2012), which summarize the parametrizations and properties of models involving density-dependence. In Rabosky (2013), Darwinian diversity-dependence and asymptotic diversity-dependence are contrasted. The former entails that a slowdown in speciation rates with diversity is in agreement with inter-species competition and Darwin’s principle of divergence. The latter represents patterns of species saturation and long term stability of diversity trajectories, but does not specify clear mechanisms of cause and effect. In general, the time scales relevant for diversity-dependence or diversity-independence are unspecified, thus leaving open whether regulatory effects act on microevolutionary rates over shorter ecological time scales, or on macroevolutionary rates averaged over much longer geological time spans (cf. Rabosky (2013). See also, Benton and Emerson (2007)).

We present here, a framework to address diversity-independent versus diversity-dependent dynamics for a family of species with a binary trait, for which the rates of creation and extinction of species as well as the rates of transition of the trait between species, are trait-dependent. Formal equivalence can be made between species level and population level dynamics, where number of species is equivalent to number of individuals, diversification rate is equivalent to growth rate, and transition in species traits is equivalent to mutation (e.g. Chevin (2016), Vellend (2010)). So, useful insights can be gained from using concepts of population genetics to species diversification analysis. We first rigorously investigate the mechanisms in species tree models which are analogous to those of population genetics models, such as mutation, selection, and genetic drift in allelic frequency models, and later, we apply the population genetics concepts to study diversity-dependent processes. The fraction of species in the family which carries one of the traits evolves in a manner directly comparable with the evolution of allele frequencies in the population genetics framework. The transition of a species of one trait to a species of the other trait resembles mutational change from one allelic type to another. Similarly, the trait-dependence present in creation rates, extinction rates, and the associated turnover rates, cause selection effects as well as frequency dependent genetic drift coefficients. Furthermore, various forms of population size dependence may be cast as density-dependent selection mechanisms. Despite, this formal equivalence, there are relevant biological differences between species level and population level models. For instance, whereas mutation is often much lower than selection in population genetics, there is no a priori reason to assume that rates of trait transition are of different magnitude than speciation and extinction rates. This makes species traits less “heritable” than individual traits. Besides, the number of species relevant to the species diversification dynamics is likely far less than the effective size of a species (100, 1000 for the former versus usually much more than $10^4$ for the latter). This makes diversification dynamics more sensitive to stochastic effects (see below).

The central technique in our approach is a diffusion approximation of a species tree Markov chain, which runs on a suitable time scale of evolutionary time units. In particular, our scaling method incorporates the point of view that “macroevolutionary speciation rates can involve processes associated with both the splitting and extinction of populations over ecological and demographic timescales”, as discussed in Rabosky (2013). From the complementary viewpoint of diffusion processes where the dynamics is provided by solutions of a PDE, our contribution relates closely to the pioneering work by Gillespie (1974). Using these links we study the effective population size in the model, and shed some light on an apparent paradoxical conclusion reported in Gillespie (1974). Several simplified, partly symmetric, parameter settings are identified in the stochastic model leading up to a reference case, or neutral case, which corresponds to fully neutral evolution in population genetics modeling.

Research has previously been carried out on similar topics, in which diffusion approximation methods were used to study density-dependent processes. Lambert (2006) provides a branching process approach to studying probability of fixation for two-allele population models, including density-dependence mechanisms. Parsons and Quince (2007a) extended a logistic growth model to a supercritical model of two competing types, and using diffusion approximation methods, estimated the probability of fixation for both types. They proposed a so called ‘non neutral’ model, in which the ratios of birth to death rates were assumed to be different for the two types, and showed that the type with higher birth to death rate ratio eventually takes over the entire population. In a subsequent analysis by Parsons and Quince (2007b), the fixation probability was again approximated using diffusion processes, but for ‘quasi neutral’ populations in which both types had equal birth to death ratios. In this case, the type with the higher birth rate shows an increase in numbers during the growth phase at densities below the carrying capacity, whereas near the carrying capacity, the type with lower birth rate is favored, due to smaller fluctuations in population density – a phenomenon termed as ‘*r* vs *K* selection’ (see e.g., Pianka (1970)). Abu Awad and Coron (2018) use a scaling approach similar to ours to study effective population mass, absorption and extinction times, etc., for a population controlled by a carrying capacity. Chevin (2016) utilized diffusion approximation methods to analyze the evolution of binary discrete and continuous traits, and interpreted diversification models in terms of population genetics concepts of species selection and random drift. In our current work, we develop a further direction by providing a thorough mathematical basis and an analytical framework for the analogy between binary trait species diversification and population genetics models. For completeness and clarity, we re-derived some of these previous results using our proposed analytical framework.

## The species branching model

We consider the dynamics of the size of a species family which carries a binary trait, marked 0 or 1, and undergoes trait-dependent splitting, extinction, and transition. We apply a two-type, continuous time Markov process $X=(X_u)_{u\ge 0}$ with components $X=(K,L)$, such that $X_u=(K_u,L_u)$ and $K_u$ and $L_u$ represent the number of species with trait 0 and trait 1, respectively, at time *u*. The two-type branching events are

1 $$\begin{aligned}&\quad \;\text{ target } \text{ state }\quad \text{ branching } \text{ rate }\nonumber \\ (k,\ell )\mapsto&\left\{ \begin{array}{cc} (k+1,\ell ) &{} \quad \lambda _0 k\\ (k,\ell +1) &{}\quad \lambda _1 \ell \\ (k-1,\ell ) &{} \quad \mu _0 k\\ (k,\ell -1) &{} \quad \mu _1 \ell \\ (k-1,\ell +1) &{} \quad \delta _{01}\, k\\ (k+1,\ell -1) &{} \quad \delta _{10}\, \ell \\ \end{array} \right. \end{aligned}$$

in terms of splitting rates $\lambda _0$, $\lambda _1$, extinction rates $\mu _0$, $\mu _1$, and transition rates $\delta _{01}$ and $\delta _{10}$. For the analysis in this work, however, it will be convenient to use the equivalent set of parameters consisting of net diversification rates $d_i$ and turnover rates $\tau _i$, $i=0,1$, given by

$$\begin{aligned} d_0=\lambda _0-\mu _0,\quad d_1=\lambda _1-\mu _1,\quad \tau _0=\lambda _0+\mu _0,\quad \tau _1=\lambda _1+\mu _1, \end{aligned}$$

in addition to the transition rates $\delta _{01}$ and $\delta _{10}$. Important aspects of the stochastic behavior of $X_u$ are determined by the eigenvalues, $\gamma _-$ and $\gamma _+$, $\gamma _-\le \gamma _+$, of the $2\times 2$ mean matrix

$$\begin{aligned} A=\left[ \begin{array}{cc} d_0-\delta _{01} &{} \delta _{10} \\ \delta _{01} &{} d_1-\delta _{10} \end{array}\right] . \end{aligned}$$

For the case $\gamma _+\le 0$ of subcritical (<) and critical ($=$) branching, the species family will go extinct at a random time $\eta _0$, the extinction time, which is finite with probability one and such that $X_u=(0,0)$ for all $u\ge \eta _0$. Under the supercritical assumption $\gamma _+>1$, with a positive probability strictly less than one the process goes extinct, otherwise the species family survives and grows in size without bound. The species model is a two-type branching process $X=(X_u)_{u\ge 0}$ with linear jump rates, such that the dynamics of $X=(K,L)'$ (here viewed as column vector) satisfy

2 $$\begin{aligned} X_u=X_0+\int _0^u AX_s\,ds+M_u,\;\; u\ge 0, \end{aligned}$$

where $X_0$ is the initial state of the model (e.g., $X_0=(1,0)'$) and $(M_u)_{u\ge 0}$ is a stochastic (martingale) term with mean ${\mathbb {E}}(M_u)=0$. A general class of two-type branching processes, including population size dependent models, is characterized in Kaj and Tahir (2019) as unique solutions of such stochastic equations.

We are particularly interested in the representation (*P*, *R*) of *X*, given by

$$\begin{aligned} P_u=\frac{K_u}{K_u+L_u},\quad R_u=K_u+L_u,\quad u\ge 0 \end{aligned}$$

so that *P* is the fraction of trait 0 among the species, and *R* is the total number of species, regardless of trait. Here, $R_u=0$, $u\ge \eta _0$, and we note that $P_u$ is well-defined for all *u*, with either $P_u=0$ or $P_u=1$ for $u\ge \eta _0$. Conversely, given (*P*, *R*), we obtain (*K*, *L*) from

$$\begin{aligned} K_u=P_uR_u,\quad L_u=(1-P_u)R_u,\quad u\ge 0. \end{aligned}$$

By using Eq. (2) and Itô’s formula for pure jump processes, one obtains a stochastic equation for (*P*, *R*), see Section 4 in Kaj and Tahir (2019).

**Fig. 1:**
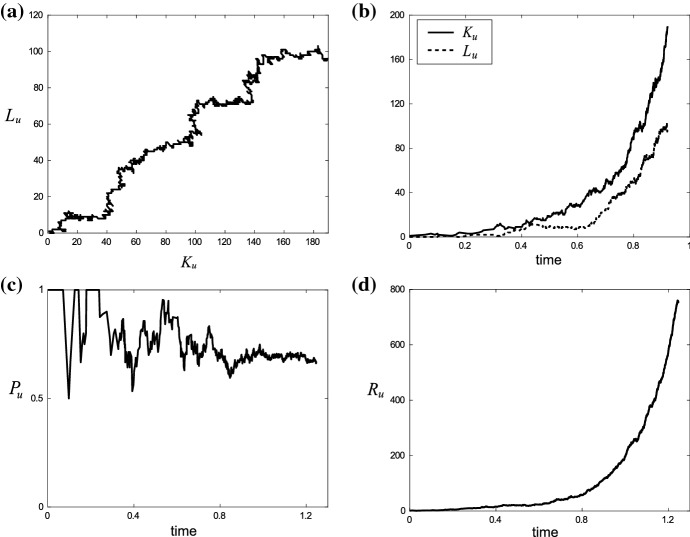
Upper panels: simulation of the two-type branching process *X*; *L* versus *K* in a) and $(K_u, L_u)$ versus time *u* in b). Lower panels: simulation of *P* versus time in c) and *R* versus time in d). Parameter values used for the simulations are $d_0=7$, $d_1=4$, $\tau _0=17$, $\tau _1=16$, $\delta _{01}=4$ and $\delta _{10}=6$

As a simple illustration, Fig. 1 shows a simulation of the two-type process $X=(K,L)$ and the corresponding representation (*P*, *R*). The choice of parameters is supercritical, such that the net growth rate is strictly positive for trait 0, $\gamma _+ = 6$, and strictly negative for trait 1, $\gamma _- = -5$. Thus, trait 1 on its own would go extinct. Here, however, the number of trait-1 species counted by *L* is sustained and growing by transitions from trait 0.

### Law of large numbers

To understand the average behavior of (*K*, *L*) and of (*P*, *R*), it is useful to consider a system which starts at time $u=0$ with *m* species, which is equivalent to summing *m* i.i.d. copies of the original model starting with one initial species each. Write $X^{(m)}=(K^{(m)},L^{(m)})'$ for such a system, with e.g. $X_0^{(m)}=(m,0)'$, and let ${{\widehat{X}}}^m=\frac{1}{m} X^{(m)}$ be the resulting average. The (strong) law of large numbers applies, so for each $u\ge 0$,

$$\begin{aligned} {{\widehat{X}}}^m_u \rightarrow \widehat{x}_u=(k_u,\ell _u)' \;\; a.s.,\quad m\rightarrow \infty , \end{aligned}$$

where the limit is the expected value ${{\widehat{x}}}_u={\mathbb {E}}(X_u)$, which solves the linear ODE

$$\begin{aligned} \frac{d}{du}{{\widehat{x}}}_u= A{{\widehat{x}}}_u, \quad {{\widehat{x}}}_0=(1,0)'. \end{aligned}$$

It follows from the law of large numbers for ${{\widehat{X}}}^m=({{\widehat{K}}}^m, {{\widehat{L}}}^m)$, that the process $(P^m,R^m)$, defined by

$$\begin{aligned} P_u^m=\frac{{{\widehat{K}}}_u^{m}}{{{\widehat{K}}}_u^{m}+\widehat{L}_u^{m}},\quad R_u^m={{\widehat{K}}}_u^{m}+{{\widehat{L}}}_u^{m},\quad u\ge 0, \end{aligned}$$

converges, as $m\rightarrow \infty$, to the solution (*p*, *r*), where $p_u=k_u/(k_u+\ell _u)$, $r_u=k_u+\ell _u$, of the deterministic limiting ODE

$$\begin{aligned} \frac{d}{du} p_u&=(d_0-d_1) p_u(1-p_u)-\delta _{01} p_u+\delta _{10}(1-p_u), \quad p_0=1\\ \frac{d}{du} r_u&=r_u\big (d_0p_u+d_1(1-p_u)\big ), \quad r_0=1. \end{aligned}$$

The limit equation for the fraction *p*, is a first indication of a connection to population genetics modeling, as it resembles the deterministic part of allele frequency dynamics (with mutation intensities $\delta _{01}$, $\delta _{10}$ and selection intensity $d_0-d_1$). In this model the net transition rate of trait 0, given by the term $-\delta _{01}p_u+\delta _{10}(1-p_u)$, arises from so called anagenetic transitions between the two types. Anagenetic transitions occur along the branches of the species tree separate from speciation events. In contrast, a cladogenetic transition is a change in trait combined with the survival of the original type, hence associated with two-type speciation. To account for cladogenetic transitions occurring with probabilities $a_0$ and $a_1$ respectively for the two types, we modify the speciation and transition rates in (1) as

$$\begin{aligned} (k,\ell )\mapsto&\left\{ \begin{array}{cc} (k+1,\ell ) &{} \quad \lambda _0 k +a_1\delta _{10}\ell \\ (k,\ell +1) &{}\quad \lambda _1 \ell +a_0\delta _{01} k\\ (k-1,\ell +1) &{} \quad (1-a_0)\delta _{01}\, k\\ (k+1,\ell -1) &{} \quad (1-a_1)\delta _{10}\, \ell \, ,\\ \end{array} \right. \end{aligned}$$

while the extinction rates remain the same (Tahir et al. 2019). Then the stated ODE for (*p*, *r*) holds with $d_0$ and $d_1$ replaced by $\tilde{d}_0=d_0+a_0\delta _{01}$ and ${{\tilde{d}}}_1=d_1+a_1\delta _{10}$. Hence the relative impact of cladogenetic transitions as measured by $a_0$ and $a_1$ affects the “selection intensity” ${{\tilde{d}}}_0-{{\tilde{d}}}_1$. This type of transition is analogous to a ‘reduction in species level heritability of the trait’ (Chevin 2016).

## Evolutionary time scaling

In addition to the short time scale of “species generations”, we introduce an evolutionary time scale and apply diffusion approximation methods to help understand the dynamics of the species model over the long time scale. Indeed, some of the most powerful tools of mathematical population genetics rely on approximation with diffusion processes, which allow for efficient computation of quantities such as fixation probabilities, expected times to fixation, and expected frequency spectra. The starting point for this approach is to relate the Markov chain *X*, with the population genetics *pre-limit* process, such as the Wright–Fisher model or Moran model, using the time scale of generations.

### Scaled Wright–Fisher and Moran models

To see the relevance of time scales, we recall the haploid version of the standard bi-allelic Wright–Fisher model in discrete time $k\ge 0$, with fixed population size *N*, selection coefficient *s* representing reproductive weights $1+s$ for trait 0 and 1 for trait 1, and probabilities $p_{01}$ and $p_{10}$ for mutations from allele 0 to 1 and 1 to 0, respectively. Letting $Z_0$ be the initial number of trait-0 alleles and $Z_k$, $k\ge 1$, the number of 0-alleles after selective sampling of *k* new generations allowing for mutational change of traits in each step, this is the Markov chain $Z=(Z_k)_{k\ge 0}$. The standard diffusion approximation of the Wright–Fisher model involves a re-scaling of both time and population size with *N*, as well as a scaling of the model parameters. For this aim, let $\gamma$, $\rho _{01}$, and $\rho _{10}$ be the scaling parameters which control the rate at which the strength of selection and mutation tends to 0 with increasing *N*, as

$$\begin{aligned} s_N=\gamma /N,\quad p_{01}^{(N)}=\rho _{01}/N,\quad p_{10}^{(N)}=\rho _{10}/N. \end{aligned}$$

More generally, these relations can be understood as limit relations, for example $s_N\sim \gamma /N$, i.e., $\lim _{N\rightarrow \infty } Ns_N=\gamma$. We write $Z^{(N)}$, for the associated scaled Wright–Fisher model, and consider the frequency process $\xi ^N=(\xi _t^N)_{t\ge 0}$ on the evolutionary time scale of *Nt* generations, defined as

$$\begin{aligned} \xi _t^{N}=\frac{1}{N}Z_{[Nt]}^{(N)}, \quad t\ge 0. \end{aligned}$$

The Wright–Fisher diffusion, $\xi =(\xi _t)_{t\ge 0}$, is the limit process of $\xi ^{N}$ as $N\rightarrow \infty$, and is known to be the unique, strong solution of the stochastic differential equation

3 $$\begin{aligned} d\xi _t=\gamma \xi _t(1-\xi _t)\,dt-\rho _{01}\xi _t\,dt +\rho _{10}(1-\xi _t)\,dt +\sqrt{\xi _t(1-\xi _t)}\,dB_t,\quad \xi _0=x,\nonumber \\ \end{aligned}$$

with *B* a Brownian motion. Written in the form $d\xi _t=b(\xi _t)\,dt + \sigma (\xi _t)\,dB_t$, with

$$\begin{aligned} b(y)=\gamma y(1-y)-\rho _{01}y +\rho _{10}(1-y),\quad \sigma ^2(y)=y(1-y), \end{aligned}$$

we may refer to *b* as the infinitesimal mean or the diffusion drift function and to $\sigma ^2$ as the infinitesimal variance, the diffusion variance, or, the genetic drift function. The Moran model applies a birth-death mechanism for reproduction of alleles, rather than the characteristic binomial sampling of the Wright–Fisher model. Since birth events, where alleles copy and spread, are always compensated for by deaths of randomly sampled individuals, the population size is still maintained at a constant level *N*. The corresponding rescaling and diffusion approximation as $N\rightarrow \infty$, yield the same limit process except for a factor 2 in the variance function, $\sigma ^2(y)=2y(1-y)$. The further parameterized case, $\sigma ^2=y(1-y)/N_\mathrm {eff}$, can be associated with the Wright–Fisher approximation of a system modulated by an effective population size, $N_\mathrm {eff}$. For the general theory we refer to Etheridge (2011).

### Gillespie approach

The infinitesimal generator of the Wright–Fisher diffusion process is the differential operator *G*, defined for a class of sufficiently regular functions *f* on the unit interval by

$$\begin{aligned} Gf(y)=\frac{1}{2}y(1-y)f''(y)+\gamma y(1-y)f'(y), \quad 0\le y\le 1, \end{aligned}$$

for which the function $u(t,x)={\mathbb {E}}_x[f(\xi _t)]$ solves Kolmogorov’s backward equation

$$\begin{aligned} \frac{\partial }{\partial t}u_t(x)=G u_t(x),\quad t>0.\quad u_0(x)=f(x),\quad 0<x<1. \end{aligned}$$

The population genetics model of Gillespie (1974) allows the population size to vary following an offspring distribution with mean $1+\mu _i$ and variance $\sigma _i^2$, for two alleles $A_i$, $i=1,2$. Writing *p* for the frequency of allele $A_1$ and *r* for the total population size, this approach suggests the two-dimensional Markov generator

4 $$\begin{aligned} Gf(p,r)&=\left( \mu _1-\mu _2+\frac{1}{r}(\sigma _2^2-\sigma _1^2)\right) \frac{\partial f}{\partial p}+\frac{p(1-p)}{r}(p\sigma _2^2+(1-p)\sigma _1^2)\frac{1}{2}\frac{\partial ^2 f}{\partial p^2}\nonumber \\&\quad + r(p\mu _1+(1-p)\mu _2)\frac{\partial f}{\partial r} +r(p\sigma _1^2+(1-p)\sigma _2^2)\frac{1}{2}\frac{\partial ^2 f}{\partial r^2}\nonumber \\&\quad + p(1-p)(\sigma _1^2-\sigma _2^2)\frac{\partial ^2 f}{\partial p\partial r}. \end{aligned}$$

Here, we are using *r* for population size as opposed to the more classical population genetics notation *n* as in Gillespie (1974), to avoid mix-up with our general scaling parameter *n* used below. The associated Kolmogorov’s backward equation is dual to the corresponding forward equation given as Equation 3 in Gillespie (1974). In particular, we read out that $r/(p\sigma _2^2+(1-p)\sigma _1^2)$ acts as a varying effective population size. It is perhaps worth stressing that this measure of effective population size varies not only with the size *r* but also, in general, with the frequency *p*, due to possibly different variance parameters $\sigma _1^2$, $\sigma _2^2$. Earlier references on random effective population size and stochastic population size models can be found in Kaj and Krone (2003), Sjödin et al. (2005), together with developments on coalescent models for such situations. An additional result in Gillespie (1974), in particular Equations 6,7, refers to the special case $\mu _1=\mu _2$ saying that the probability of fixation of allele $A_1$, given an initial frequency *p*, equals

5 $$\begin{aligned} u(p)= \frac{\sigma _2^2 p}{\sigma _1^2(1-p)+\sigma _2^2 p} \end{aligned}$$

The diffusion approximation model of species richness we develop next will be closely related to the model described by Eq. (4). We will recover and discuss further the role of both the random effective population size $r/(p\sigma _2^2+(1-p)\sigma _1^2)$ and the fixation probability *u*(*p*).

### Scaled species model

Our investigation takes the view that, given non-extinction of the continuous time branching process *X*, the component $K=(K_u)_{u\ge 0}$ in the species model relates naturally to the discrete time Markov chain $(Z_k)_{k\ge 0}$, of the population genetics model. Of course these quantities are not immediately comparable since the total number of species *R* varies randomly, while the population size *N* is fixed from one generation to the next. For this reason, we introduce a species family process $X^{(n)}=(K^{(n)},L^{(n)})'$ marked by a separate scaling variable *n*. The two-type branching process is driven by an initial number of species of magnitude *n*, and has scaled parameters $d_0^{(n)}$, $d_1^{(n)}$, $\tau _0^{(n)}$, $\tau _1^{(n)}$, $\delta _{01}^{(n)}$ and $\delta _{10}^{(n)}$, which control the rates of diversification, turnover, and transition on the population timescale. For fixed *t*, the fraction $n^{-1} X_{[nt]}^{(n)}$, is the typical configuration of trait frequencies in the species family seen over the span of *t* time units that elapse upon completing [*nt*] generations. The precise behavior of the rates as functions of *n* is not crucial as long as they scale for large *n* with the following macroevolutionary rates which act on the new time scale,

6 $$\begin{aligned} d_i^{(n)}\sim \frac{\beta _i}{n},\quad \tau _i^{(n)}\sim \tau _i,\quad i=0,1 \qquad \delta _{01}^{(n)}\sim \frac{\rho _{01}}{n},\quad \delta _{10}^{(n)}\sim \frac{\rho _{10}}{n}. \end{aligned}$$

Here, $\tau _i$ measures variance in the production of daughter species and is the reference turnover parameter for extinction and/or speciation events of trait *i*, $\beta _i$ is the macroevolutionary net diversification rate of trait *i*, seen as the long-time average net effect of speciation and extinction, and $\rho _{01}$ and $\rho _{10}$ are the resulting macroevolutionary rates of exchange of traits. For later reference we also adopt from Gillespie (1977) the *new evolutionary principle* that trait success (fitness in population genetics terminology) not only increases with offspring mean but also may decrease with offspring variance inversely proportional to population size. Using macroevolution terminology, $\tau$ refers to the species turn-over rate, a quantity which has received increasing attention in the empirical literature aiming at interpreting patterns of species diversity (Gamisch and Comes 2019; Han et al. 2020; Nakov et al. 2019). In the current situation of developmental stochastic dynamics, as opposed to environmental stochastic influences, $\beta _i$ (or rather $1+\beta _i$) represent offspring mean and $\tau _i$ offspring variance. Then, following Gillespie (1974, 1977) the fitness is properly measured by $f_i(r)$, where

7 $$\begin{aligned} f_i(r)=\beta _i-\frac{\tau _i}{r},\quad i=0,1. \end{aligned}$$

Conditionally on the set of paths of *X* which do not go extinct, let

$$\begin{aligned} P^n_t = \frac{K_{nt}^{(n)}}{K^{(n)}_{nt}+L^{(n)}_{nt}}, \qquad R^n_t=\frac{1}{n} (K^{(n)}_{nt}+L^{(n)}_{nt}), \quad t\ge 0. \end{aligned}$$

The ratio $P^n$ will play the role of the frequency process $\xi ^N$, even though the scaled Wright Fisher process $\xi ^N$ is derived from a population of fixed size *N* whereas $P^n$ is a frequency with respect to the stochastically varying species richness process $R^n$.

The final step towards a generic species trait richness model is finding the limit processes in the scaling regime as $n\rightarrow \infty$, expecting the remaining macroevolutionary rates to be $\tau _0$, $\tau _1$ as rates of change for the speciation/extinction process, $\beta _0$, $\beta _1$ diversification rates for net growth (if positive) or net decline (if negative), and $\rho _{01}$, $\rho _{10}$ controlling trait interchange. We expect in the limit to obtain a bi-variate, coupled diffusion process, ${\mathcal {X}}=({\mathcal {X}}_t)_{t\ge 0}$, with representations $({\mathcal {K}},{\mathcal {L}})'$ and $({\mathcal {P}},{\mathcal {R}})$ say. To assist in identifying the limit model we will exploit the existing theory of measure-branching processes, briefly laid out in Sect. 5 below. In fact, it is straightforward to re-interpret $X^{(n)}$ as a measure-valued branching process on trait space $E=\{0,1\}$ with scaled parameters. In this view our macroevolutionary scaling is consistent with a super-process approximation, for which the spatial motion is the on-off process on *E* with jump rates $\rho _{01}$, $\rho _{10}$. As a consequence, $(n^{-1}X_{[nt]}^{(n)})_{t\ge 0}$, has a weak limit which is a super-on/off process with binary branching.

#### Proposition 1

Suppose that

$$\begin{aligned} n^{-1}X^{(n)}_0\rightarrow {\mathcal {X}}_0,\quad n\rightarrow \infty , \end{aligned}$$

where ${\mathcal {X}}_0$ is a nonnegative deterministic column vector. As $n\rightarrow \infty$ the scaled branching process $(n^{-1}X_{nt}^{(n)})_{t\ge 0}$, converges weakly to the continuous state branching process ${\mathcal {X}}=({\mathcal {K}},{\mathcal {L}})'$, which is the unique solution of the SDE

$$\begin{aligned} {\mathcal {X}}_t&= {\mathcal {X}}_0+ \int _0^t \left[ \begin{array}{cc} \beta _0-\rho _{01} &{} \rho _{10} \\ \rho _{01} &{} \beta _1-\rho _{10} \end{array} \right] {\mathcal {X}}_s\,ds +\left( {\begin{array}{c}1\\ 0\end{array}}\right) \int _0^t \sqrt{\tau _0 {\mathcal {K}}_s} \,dB^0_s \\&\quad +\left( {\begin{array}{c}0\\ 1\end{array}}\right) \int _0^t \sqrt{\tau _1 {\mathcal {L}}_s} \,dB^1_s,\quad t\ge 0, \end{aligned}$$

where $B^0$, $B^1$ are independent, standard Brownian motions. Furthermore, $(P^n, R^n)$ converges weakly to $({\mathcal {P}},{\mathcal {R}})$, the unique solution of

8 $$\begin{aligned} d{\mathcal {P}}_t&={\mathcal {P}}_t(1-{\mathcal {P}}_t) (f_0({\mathcal {R}}_t)-f_1({\mathcal {R}}_t))\,dt -\rho _{01}{\mathcal {P}}_t\,dt+\rho _{10}(1-{\mathcal {P}}_t)\,dt\nonumber \\&\quad + \sqrt{{\mathcal {P}}_t\big (1-{\mathcal {P}}_t\big )\big (\tau _0(1-{\mathcal {P}}_t)+\tau _1 {\mathcal {P}}_t\big )\frac{1}{{\mathcal {R}}_t}}\,dB^-_t, \end{aligned}$$

9 $$\begin{aligned} d{\mathcal {R}}_t&={\mathcal {R}}_t\big (\beta _0 {\mathcal {P}}_t+\beta _1(1-{\mathcal {P}}_t)\big )\,dt +\sqrt{{\mathcal {R}}_t\big (\tau _0 {\mathcal {P}}_t+\tau _1(1-{\mathcal {P}}_t)\big )}\,dB^+_t, \end{aligned}$$

where $f_0(r)$ and $f_1(r)$ are the fitness measures in Eq. (7) and $B^-_t$, $B^+_t$ are standard Brownian motions. The Brownian motions are independent when $\tau _0=\tau _1$. In general, the quadratic covariation processes of $(B^-,B^+)$ and $({\mathcal {P}},{\mathcal {R}})$ are given by

$$\begin{aligned} \langle \!\langle B^-,B^+\rangle \!\rangle _t =\Big (\frac{\tau _0}{\tau _1}-1\Big ) \int _0^t \frac{\sqrt{{\mathcal {P}}_s(1-{\mathcal {P}}_s)}}{\sqrt{\big (\tau _0(1-{\mathcal {P}}_s)/ \tau _1+{\mathcal {P}}_s\big )\big (\tau _0{\mathcal {P}}_s/\tau _1+1-{\mathcal {P}}_s\big )}}\,ds, \end{aligned}$$

and

10 $$\begin{aligned} \langle \!\langle {\mathcal {P}},{\mathcal {R}}\rangle \!\rangle _t=(\tau _0-\tau _1) \int _0^t {\mathcal {P}}_s(1-{\mathcal {P}}_s)\,ds. \end{aligned}$$

### Effective population size: resolving Gillespie’s paradox

The infinitesimal mean and infinitesimal variance terms of the diffusions ${\mathcal {P}}$ and ${\mathcal {R}}$, except for the mutation terms involving $\rho _{01}$ and $\rho _{10}$, as well as the covariation in Eq. (10), have matching terms in the generator *G* in Eq. (4). In Eq. (8) we recognize a Wright–Fisher diffusion with selection and mutation, and with trait-dependent genetic drift (or ‘species drift’) ${\mathcal {P}}_t(1-{\mathcal {P}}_t)/{\mathcal {N}}_t$ in the diffusion variance of $B^-$, such that

11 $$\begin{aligned} {\mathcal {N}}_t=\frac{{\mathcal {R}}_t}{\tau _0(1-{\mathcal {P}}_t)+\tau _1{\mathcal {P}}_t} \end{aligned}$$

acts as a stochastically varying effective population size for the differential dynamics of $d{\mathcal {P}}_t$. This observation is parallel to that in the original work Gillespie (1974), where the corresponding quantity appears in the coefficient of $\partial ^2 f/\partial p^2$ in Eq. (4). The species drift increases as the number of species decreases, analogous to genetic drift of the allele frequency in population genetics. Similarly, the species drift increases with the turn over rates $\tau _0$ and $\tau _1$, which represent the species offspring variance. By applying Ito’s formula to ${\mathcal {N}}_t=g({\mathcal {P}}_t,{\mathcal {R}}_t)$, $t\ge 0$, with $g(p,r)=r/(\tau _0(1-p)+\tau _1p)$, we obtain directly the dynamics of $({\mathcal {P}},{\mathcal {N}})$ as an alternative to that of $({\mathcal {P}},{\mathcal {R}})$. For simplicity, we consider the case where the diversification rates are equal, $\beta _0=\beta _1=\beta$, and the rates of trait exchange vanish, $\rho _{01}=\rho _{10}=0$. Then

$$\begin{aligned} d{\mathcal {P}}_t&= -\frac{(\tau _0-\tau _1){\mathcal {P}}_t(1-{\mathcal {P}}_t)}{\tau _0(1-{\mathcal {P}}_t)+\tau _1{\mathcal {P}}_t} \frac{1}{{\mathcal {N}}_t}\,dt + \sqrt{{\mathcal {P}}_t\big (1-{\mathcal {P}}_t\big ) \frac{1}{{\mathcal {N}}_t}}\,dB^-_t,\\ d{\mathcal {N}}_t&=\beta {\mathcal {N}}_t\,dt +\frac{(\tau _0-\tau _1)^2{\mathcal {P}}_t(1-{\mathcal {P}}_t)}{(\tau _0(1-{\mathcal {P}}_t)+\tau _1{\mathcal {P}}_t)^2} \,dt\\&\quad +\sqrt{{\mathcal {N}}_t \frac{\tau _0 {\mathcal {P}}_t+\tau _1(1-{\mathcal {P}}_t)}{\tau _0(1-{\mathcal {P}}_t)+\tau _1{\mathcal {P}}_t}}\,dB^+_t + \sqrt{{\mathcal {N}}_t \frac{(\tau _0-\tau _1){\mathcal {P}}_t(1-{\mathcal {P}}_t)}{\tau _0(1-{\mathcal {P}}_t)+\tau _1{\mathcal {P}}_t}}\,dB_t^- \end{aligned}$$

The benefit of this additional representation of ${\mathcal {P}}$ in comparison with the previous stochastic equation (8), is that the effective population size ${\mathcal {N}}$ in the species drift of ${\mathcal {P}}$ now appears in classical form. The disadvantage is the increased complexity of the diffusion drift for ${\mathcal {P}}$ as well as diffusion drift and variance of ${\mathcal {N}}$, as compared to those of ${\mathcal {R}}$ in (9).

The discussion in Gillespie (1974) also points to a potential drawback of the appearance of ${\mathcal {N}}$ in the present form in (11). If trait 1 is rare or near elimination from the species family, so that ${\mathcal {P}}\approx 1$, then formally ${\mathcal {N}}\approx {\mathcal {R}}/\tau _1$ which suggests that the now-absent trait 1 controls the effective size through the parameter $\tau _1$. Quoting Gillespie (1974): *This rather uncomfortable conclusion suggests that the concept of effective population size loses a good deal of its value in the context of the present model*. Using the wider frame of linked diffusion processes, however, this apparent paradoxical conclusion may be avoided. The above relation for $d{\mathcal {N}}_t$ suggests that if trait 1 would be eliminated and hence ${\mathcal {P}}$ reach fixation at 1, then the resulting effective size $({\mathcal {N}}_t^0)_{t\ge 0}$, would satisfy

$$\begin{aligned} d{\mathcal {N}}^0_t =\beta {\mathcal {N}}^0_t\,dt +\sqrt{{\mathcal {N}}^0_t (\tau _0/\tau _1)}\,dB^+_t, \end{aligned}$$

and hence depend on the relative turnover ratio $\tau _0/\tau _1$ and $\beta$, rather than just $\tau _1$. Of course, for the same parameter settings, if we let ${\mathcal {P}}$ tend to 1 in (9), the resulting richness ${\mathcal {R}}_t^0$ of trait 0 satisfies

12 $$\begin{aligned} d{\mathcal {R}}^0_t =\beta {\mathcal {R}}^0_t\,dt +\sqrt{{\mathcal {R}}^0_t \tau _0}\,dB^+_t, \end{aligned}$$

consistent with the relation ${\mathcal {N}}^0={\mathcal {R}}^0/\tau _1$, which appears to have been the origin of the “uncomfortable conclusion” discussed in Gillespie (1974). The diffusions ${\mathcal {N}}^0$ and ${\mathcal {R}}^0$, are Feller processes with linear drift belonging to the larger class of continuous state branching processes. It is a classical result in branching process theory that, if ${\mathcal {R}}_0^0=r$, and accordingly ${\mathcal {N}}_0^0=r/\tau _1$, then the extinction probabilities are given by

$$\begin{aligned} {\mathbb {P}}_r(\lim _{t\rightarrow \infty }{\mathcal {R}}_t^0=0)=e^{-2r\beta /\tau _0} \end{aligned}$$

and

$$\begin{aligned} {\mathbb {P}}_{r/\tau _1}(\lim _{t\rightarrow \infty }{\mathcal {N}}_t^0=0)=e^{-2(r/\tau _1)\cdot \beta /(\tau _0/\tau _1)} =e^{-2r\beta /\tau _0}. \end{aligned}$$

Naturally, they no more depend on the parameter $\tau _1$. In response to a question raised by an anonymous referee, we also mention an alternative approach to this topic. By changing variables from (*p*, *r*) to (*p*, *n*) with $n=r/(\sigma ^2_1(1-p)+\sigma ^2_2 p)$ one obtains a new generator *Gf*(*p*, *n*) parallel to *Gf*(*p*, *r*) in (4). The coefficients in *Gf*(*p*, *n*) will now have matching terms to those in the SDE for $({\mathcal {P}}_t,{\mathcal {N}}_t)$.

### Further interpretation of Eqs. (8–10)

The species ‘selection coefficient’ in Eq. (8) is the fitness difference

$$\begin{aligned} \gamma _t=f_0({\mathcal {R}}_t)-f_1({\mathcal {R}}_t)=\beta _0-\beta _1-\frac{\tau _0-\tau _1}{{\mathcal {R}}_t}, \quad t\ge 0, \end{aligned}$$

dependent on net diversification rates, turnover rates and species richness. If $\beta _0>\beta _1$, so that trait 0 has larger net diversification, then trait 0 has a “fitness” advantage over trait 1, $\gamma _t>0$ at time *t*, if either $\tau _1>\tau _0$ or $\tau _0>\tau _1$ and ${\mathcal {R}}_t>(\tau _0-\tau _1)/(\beta _0-\beta _1)$. In other words, species-level selection is reduced in small populations and enhanced in large. If $\tau _1>\tau _0$, the opposite holds. Hence, traits with higher diversification rates and slower turnover rates are favored by species selection. The time average of $\gamma$ up to time *t* is

$$\begin{aligned} \frac{1}{t}\int _0^t \gamma _s\,ds = (\beta _0-\beta _1) (1-F_t), \end{aligned}$$

where

$$\begin{aligned} F_t=\frac{\tau _0-\tau _1}{\beta _0-\beta _1} \frac{1}{t} \int _0^t \frac{1}{{\mathcal {R}}_s} \,ds \end{aligned}$$

can be seen as the relative contribution of $\tau _0-\tau _1$ to species selection (Chevin 2016).

The total species richness is measured by ${\mathcal {R}}$ in Eq. (9), which is a continuous state branching process with linear drift and trait-dependence in both the diffusion drift and variance functions. Conditional on non-extinction, ${\mathcal {R}}$ will grow exponentially as $t\rightarrow \infty$. Hence, the diffusion variance term for $d{\mathcal {P}}_t$ in (8), driven by $B^-$, vanishes. The diffusion drift function for $d{\mathcal {P}}_t$ also simplifies and we expect ${\mathcal {P}}_t\rightarrow p_\infty$ as $t\rightarrow \infty$, where $p_\infty$ is the solution of the second order equation

13 $$\begin{aligned} p_\infty (1-p_\infty )(\beta _0-\beta _1)- \rho _{01}p_\infty +\rho _{10}(1-p_\infty )=0. \end{aligned}$$

The covariation of $${\mathcal {P}}$$ and $${\mathcal {R}}$$ in Eq. (10) measures the degree to which these quantities vary simultaneously, in the sense that the expected change over a small time interval $$[t,t+h)$$, conditional on the present state $$({\mathcal {P}}_t,{\mathcal {R}}_t)$$, is given by

$$\begin{aligned} {\mathbb {E}}[({\mathcal {P}}_{t+h}-{\mathcal {P}}_t)({\mathcal {R}}_{t+h}-{\mathcal {R}}_t)|({\mathcal {P}}_t,{\mathcal {R}}_t)] =(\tau _0-\tau _1){\mathcal {P}}_t(1-{\mathcal {P}}_t)h+o(h),\quad h\rightarrow 0. \end{aligned}$$

We observe that, while the covariation of ${\mathcal {P}}$ and ${\mathcal {R}}$ is proportional to the difference in turnover rates, $\tau _0-\tau _1$, the analogous covariation of the two underlying Brownian motions, $B^-$ driving ${\mathcal {P}}$ and $B^+$ driving ${\mathcal {R}}$, is a function only of the turnover-ratio $\tau _0/\tau _1$. In both cases, the dependence is positive in case $\tau _0>\tau _1$ and negative otherwise. We conclude that the species richness co-varies positively with the trait with the highest turnover. The turnover ratio $\tau _0/\tau _1$ determines the strength of the covariation which is intrinsic at the level of Brownian noise, whereas both of $\tau _0$ and $\tau _1$ are required in order to find the covariation of the state variables ${\mathcal {P}}$ and ${\mathcal {R}}$.

### Special cases

#### (a) Symmetric net diversification rates, $\beta _0=\beta _1$

Let $\beta _0=\beta _1=\beta$. Then, from Eq. (8) and Eq. (9), we have

$$\begin{aligned} d{\mathcal {P}}_t&={\mathcal {P}}_t(1-{\mathcal {P}}_t)\frac{-(\tau _0-\tau _1)}{{\mathcal {R}}_t}\,dt -\rho _{01}{\mathcal {P}}_t\,dt+\rho _{10}(1-{\mathcal {P}}_t)\,dt\\&\quad + \sqrt{{\mathcal {P}}_t\big (1-{\mathcal {P}}_t\big )\big (\tau _0(1-{\mathcal {P}}_t)+\tau _1 {\mathcal {P}}_t\big )\frac{1}{{\mathcal {R}}_t}}\,dB^-_t,\\ d{\mathcal {R}}_t&=\beta {\mathcal {R}}_t\,dt +\sqrt{{\mathcal {R}}_t\big (\tau _0 {\mathcal {P}}_t+\tau _1(1-{\mathcal {P}}_t)\big )}\,dB^+_t. \end{aligned}$$

By Eq. (10) the covariance structure remains the same as in the general model. Here, ${\mathcal {R}}$ is a supercritical Feller-type diffusion process with diffusion variance function modulated by the trait proportions ${\mathcal {P}}$ and $1-{\mathcal {P}}$. The SDE for ${\mathcal {P}}$ can be viewed as a Wright–Fisher diffusion with population size dependent infinitesimal drift and variance functions. The selection coefficient $-(\tau _0-\tau _1)/{\mathcal {R}}$ shows that the species associated with the trait of the smallest of the two turnover rates, i.e. trait 0 if $\tau _0<\tau _1$ and trait 1 if $\tau _0>\tau _1$, are selected for instead of species with the higher turnover. Biologically, this means that, all other things being equal, more long-lived species are selected for, as increasing the species generation time will automatically reduce $\tau$ (Lin et al. 2012).

#### (b) Symmetric turnover rates, $\tau _0=\tau _1$

Let $\tau _0=\tau _1=\tau$. Then Eq. (8) and Eq. (9) simplify as

$$\begin{aligned} d{\mathcal {P}}_t&={\mathcal {P}}_t(1-{\mathcal {P}}_t) (\beta _0-\beta _1)\,dt -\rho _{01}{\mathcal {P}}_t\,dt+\rho _{10}(1-{\mathcal {P}}_t)\,dt \\&\qquad + \sqrt{\frac{\tau {\mathcal {P}}_t(1-{\mathcal {P}}_t)}{{\mathcal {R}}_t}}\,dB^-_t,\\ d{\mathcal {R}}_t&={\mathcal {R}}_t\big (\beta _0 {\mathcal {P}}_t+\beta _1(1-{\mathcal {P}}_t)\big )\,dt +\sqrt{\tau {\mathcal {R}}_t}\,dB^+_t, \end{aligned}$$

with independent Brownian motions $B^-$ and $B^+$. The equation for ${\mathcal {P}}$ is the SDE of the Wright–Fisher diffusion process with selection coefficient $\beta _0-\beta _1$ and mutation rates $\rho _{01}$ and $\rho _{10}$ as in Eq. (3), except that now the genetic drift term is inversely proportional to ${\mathcal {R}}$, which acts as a randomly varying effective population size. At the same time, the population richness process ${\mathcal {R}}$ is a Feller-type diffusion process with diffusion drift function modulated by the trait proportions ${\mathcal {P}}$ and $1-{\mathcal {P}}$.

#### (c) Neutral evolution: symmetric net diversification rates, $\beta _0=\beta _1$ and turn over rates, $\tau _0=\tau _1$

Let $\tau _0=\tau _1=\tau$ and $\beta _0=\beta _1=\beta$ be nonnegative parameters. By combining the cases a) and b), we obtain

$$\begin{aligned} d{\mathcal {P}}_t&= -\rho _{01}{\mathcal {P}}_t\,dt+\rho _{10}(1-{\mathcal {P}}_t)\,dt + \sqrt{\frac{\tau {\mathcal {P}}_t(1-{\mathcal {P}}_t)}{{\mathcal {R}}_t}}\,dB^-_t,\\ d{\mathcal {R}}_t&=\beta {\mathcal {R}}_t\,dt +\sqrt{\tau {\mathcal {R}}_t}\,dB^+_t. \end{aligned}$$

Here, the first equation of ${\mathcal {P}}$ is comparable to the Wright–Fisher diffusion process Eq. (3) with only mutation and no selection, hence the term ‘neutral evolution’. The total size process is a supercritical Feller diffusion process, which we encountered previously in (12) as a limiting case under trait fixation. In the present context, we use once more that for a given initial size ${\mathcal {R}}_0=r$, the extinction probability that ${\mathcal {R}}_t\rightarrow 0$ as $t\rightarrow \infty$, equals $e^{-2\beta r/\tau }$. From this we retrieve the known result in stochastic demography that higher turn-over rate increases the extinction risk. On the complementary set of nonextinction, the total size process tends to infinity almost surely, ${\mathcal {R}}_t\rightarrow \infty$ a.s.

#### (d) Quasi-neutral rates

Quasi-neutrality, as proposed and discussed in Parsons and Quince (2007b), Parsons et al. (2008), is a rate symmetry condition, which in our notation would mean

$$\begin{aligned} \frac{\tau _0}{\beta _0} =\frac{\tau _1}{\beta _1}= \kappa . \end{aligned}$$

According to Parsons et al. (2008), the ratio $2\beta _i/\tau _i$ is an alternative measure of relative success or relative fitness of trait *i*. In this framework, therefore, quasi-neutral traits are equally fit. Eq. (8) and Eq. (9) become

$$\begin{aligned} d{\mathcal {P}}_t&={\mathcal {P}}_t(1-{\mathcal {P}}_t)\Big ((\beta _0-\beta _1)\big (1-\frac{\kappa }{{\mathcal {R}}_t}\big )\Big )\,dt -\rho _{01}{\mathcal {P}}_t\,dt+\rho _{10}(1-{\mathcal {P}}_t)\,dt\\&\quad + \sqrt{{\mathcal {P}}_t\big (1-{\mathcal {P}}_t\big )\big (\beta _0(1-{\mathcal {P}}_t)+\beta _1 {\mathcal {P}}_t\big )\frac{\kappa }{{\mathcal {R}}_t}}\,dB^-_t,\\ d{\mathcal {R}}_t&={\mathcal {R}}_t\big (\beta _0 {\mathcal {P}}_t+\beta _1(1-{\mathcal {P}}_t)\big )\,dt +\sqrt{\kappa {\mathcal {R}}_t\big (\beta _0 {\mathcal {P}}_t+\beta _1(1-{\mathcal {P}}_t)\big )}\,dB^+_t. \end{aligned}$$

## Application of the population genetics approach to diversity-dependent models

We discuss the effect of diversity dependence on the species richness model. The interaction is imposed by letting the instantaneous rates of speciation and extinction in (6) depend on the scaled total size $R^n$. A variety of interaction schemes can be incorporated into the measure-branching model formalism, which we use to derive Proposition 1. For an introduction to some of the principles of interaction in measure-branching and superprocesses, see Etheridge (2000). The relevant approach for us is the use of an analogue of the Girsanov theorem that allows for the introduction of “non-linear branching”, and in the end logistic versions of the superprocess limits obtained by an absolutely continuous change-of-drift. Our analysis is therefore similar in many ways to the development of the spatial model in Fournier and Méléard (2004), considering our trait space as the mark of location. As in Fournier and Méléard (2004) we apply superprocess techniques but at this stage our interaction schemes are less general. It is also known that logistic Feller diffusion processes arise under much more general branching mechanisms than ours (Lambert 2005), indicating more general results.

Suppose the current state of the species model at time *t* is $(P_t^n,R_t^n)$. A constant *c* will have the role of a ‘carrying capacity’. We consider in Sect. 4.1 the diversity-dependent species models which have jump rates at *t*, given by

$$\begin{aligned} \tau _i^{(n)}= \tau _i,\quad d_i^{(n)}= \frac{\beta _i}{n}\Big (1-\frac{R_t^n}{c}\Big ). \end{aligned}$$

- In greater generality, the carrying capacity is determined by a pair of parameters, $c_0$, $c_1$, and the strength in reduction of the net growth is trait-dependent. In Sect. 4.2 we consider the corresponding model with jump rates

$$\begin{aligned} \tau _i^{(n)}= \tau _i,\quad d_i^{(n)}= \frac{\beta _i}{n}\Big (1-\frac{R_t^n}{c_i}\Big ),\quad i=0,1. \end{aligned}$$

Further generalization can also be made by assuming that the two kinds of species both affect and are affected differently by species richness:

$$\begin{aligned} d_i^{(n)}= \frac{\beta _i}{n}\left( 1- R_t^n \left( \frac{P_t^n}{c_{0i}}+\frac{1-P_t^n}{c_{1i}}\right) \right) ,\quad i=0,1. \end{aligned}$$

This parallels Lotka-Volterra competition models but this latter case will not be developed further here and is left for future work. We have applied classical logistic type diversity-dependence acting on the macroevolutionary net growth rate $nd_i^{(n)}$. Starting from $R_0^n<c$, the net effect of speciations and extinctions is reduced with increasing species richness and turns into a subcritical regime above the level *c*, in case $R_t^n>c$.

To simplify notation in the rest of this section we introduce the function

14 $$\begin{aligned} \psi (p)=\tau _0(1-p)+\tau _1 p, \quad 0\le p\le 1. \end{aligned}$$

### Diversity-dependent macroevolutionary diversification rates

The limit process as $n\rightarrow \infty$ is obtained as in Proposition 1, with $\beta _0$ and $\beta _1$ at time *t* replaced by the species richness dependent functions $\beta _0 (1-{\mathcal {R}}_t/c)$ and $\beta _1 (1-{\mathcal {R}}_t/c)$. The system in Eqs. (8)–(9) becomes

15 $$\begin{aligned} d{\mathcal {P}}_t&={\mathcal {P}}_t(1-{\mathcal {P}}_t)\big (f^c_0({\mathcal {R}}_t)-f^c_1({\mathcal {R}}_t) \big )\,dt\nonumber \\&\quad -\rho _{01}{\mathcal {P}}_t\,dt+\rho _{10}(1-{\mathcal {P}}_t)\,dt + \sqrt{ {\mathcal {P}}_t(1-{\mathcal {P}}_t) \psi ({\mathcal {P}}_t) \frac{1}{{\mathcal {R}}_t}}\,dB^-_t,\\ d{\mathcal {R}}_t&={\mathcal {R}}_t \Big (1-\frac{{\mathcal {R}}_t}{c}\Big ) \big (\beta _0 {\mathcal {P}}_t+\beta _1(1-{\mathcal {P}}_t)\big )\,dt +\sqrt{{\mathcal {R}}_t \psi (1-{\mathcal {P}}_t)}\,dB^+_t, \nonumber \end{aligned}$$

with trait fitness now of the form

$$\begin{aligned} f_i^c(r)= \beta _i\Big (1-\frac{r}{c}\Big ) -\frac{\tau _i}{r}. \end{aligned}$$

In particular, if we impose the additional assumptions of neutral rates discussed as special case c) in Sect. 3.6, the process ${\mathcal {R}}$ is the solution of

$$\begin{aligned} d{\mathcal {R}}_t = \beta {\mathcal {R}}_t (1-{\mathcal {R}}_t/c)\,dt +\sqrt{\tau {\mathcal {R}}_t}\,dB^+_t,\quad t\ge 0. \end{aligned}$$

This is the logistic Feller diffusion process, which has non-negative paths and goes extinct with probability one. The logistic Feller diffusion is known to have a quasi-stationary distribution in the sense of a Yaglom limit, namely that the law of ${\mathcal {R}}_t$ conditioned on $\{{\mathcal {R}}_t>0\}$ converges as $t\rightarrow \infty$ to a probability measure (Cattiaux et al. 2009). Such a Yaglom limit, however, is not known in explicit terms and need not even be unique. For our model, we note that Cattiaux et al. (2009) Thm 8.2, yields uniqueness of the Yaglom limit for the case $c<1$, whereas under biologically relevant conditions we expect $c>1$.

For the general, non-neutral, case, letting ${{\widetilde{{\mathcal {R}}}}}={\mathcal {R}}/c$ be the total species richness in relation to the carrying capacity *c*, the SDE system for the state variables is

$$\begin{aligned} d{\mathcal {P}}_t&={\mathcal {P}}_t(1-{\mathcal {P}}_t)\Big ((\beta _0-\beta _1)(1-{{\widetilde{{\mathcal {R}}}}}_t) -\frac{\tau _0-\tau _1}{c{{\widetilde{{\mathcal {R}}}}}_t}\Big )\,dt \\&\quad -\rho _{01}{\mathcal {P}}_t\,dt+\rho _{10}(1-{\mathcal {P}}_t)\,dt + \sqrt{ {\mathcal {P}}_t(1-{\mathcal {P}}_t)\psi ({\mathcal {P}}_t)\frac{1}{c{{\widetilde{{\mathcal {R}}}}}_t}} \,dB^-_t,\\ d{{\widetilde{{\mathcal {R}}}}}_t&={{\widetilde{{\mathcal {R}}}}}_t (1-{{\widetilde{{\mathcal {R}}}}}_t)\big (\beta _0 {\mathcal {P}}_t+\beta _1(1-{\mathcal {P}}_t)\big )\,dt +\sqrt{\frac{1}{c} {{\widetilde{{\mathcal {R}}}}}_t \psi (1-{\mathcal {P}}_t)}\,dB^+_t. \end{aligned}$$

The two SDEs with respect to $B^-$ and $B^+$ have second order moments proportional to 1/*c*. This can be used to show formally that the diffusion terms vanish in the subsequent limit of large carrying capacity, $c\rightarrow \infty$, revealing the deterministic limit equation

$$\begin{aligned} d{\mathcal {P}}_t&={\mathcal {P}}_t(1-{\mathcal {P}}_t)(\beta _0-\beta _1)(1-{{\widetilde{{\mathcal {R}}}}}_t)\,dt -\rho _{01}{\mathcal {P}}_t\,dt+\rho _{10}(1-{\mathcal {P}}_t)\,dt,\\ d{{\widetilde{{\mathcal {R}}}}}_t&={{\widetilde{{\mathcal {R}}}}}_t \,(1-{{\widetilde{{\mathcal {R}}}}}_t) \big (\beta _0 {\mathcal {P}}_t+\beta _1(1-{\mathcal {P}}_t)\big )\,dt, \end{aligned}$$

that is, the ODE system

$$\begin{aligned} p_t'&=p_t(1-p_t) (1-{{\tilde{r}}}_t) (\beta _0-\beta _1) -\rho _{01}p_t+\rho _{10}(1-p_t),\\ {{\tilde{r}}}_t'&={{\tilde{r}}}_t (1-{{\tilde{r}}}_t) \big (\beta _0 p_t+\beta _1(1-p_t)\big ). \end{aligned}$$

With initial value $(p_0, {{\tilde{r}}}_0)\in (0,1)^2$, the equilibrium solution as *t* tends to infinity, is $p_\infty = \rho _{10}/(\rho _{10}+\rho _{01})$, ${{\tilde{r}}}_\infty = 1$. We see that species selection, acting on the net diversification rates $\beta _0$ and $\beta _1$, is effective only during the growth phase but vanishes as the number of species approaches the carrying capacity, i.e., when ${{\tilde{r}}}_t$ approaches 1. In ecology, this is said to be a model with only *r*-selected traits but no *K*-selected traits (where *r* is the growth rate and *K* is the carrying capacity), that is, the diversification of the two traits is regulated in the same way as ${{\tilde{r}}}_t$ approaches 1 (Pianka 1970). This scenario would correspond to a trait allowing adaptive radiation with a rapid diversification of species possessing this trait when many niches are still available, until an almost neutral dynamics when carrying capacity is reached.

### Trait- and diversity-dependent macroevolutionary diversification rates

The density dependent mechanism is again to reduce the growth of the species family with increasing diversity, but now the efficiency of the nonlinear influence is tuned for each trait, with the resulting trait fitness

$$\begin{aligned} f_i^{c_i}(r)= \beta _i(1-r/c_i)-\tau _i/r, \quad i=0,1. \end{aligned}$$

By adapting the derivation leading to Eq. (8)–(9) to this case and putting ${{\widetilde{{\mathcal {R}}}}}={\mathcal {R}}/c$ as before, then in the limit $n\rightarrow \infty$,

$$\begin{aligned} d{\mathcal {P}}_t&={\mathcal {P}}_t(1-{\mathcal {P}}_t)\Big (f_0^{c_0}(c\widetilde{\mathcal {R}}_t)-f_1^{c_1}(c{{\widetilde{{\mathcal {R}}}}}_t)\Big )\,dt -\rho _{01}{\mathcal {P}}_t\,dt+\rho _{10}(1-{\mathcal {P}}_t)\,dt\\&\quad + \sqrt{ {\mathcal {P}}_t(1-{\mathcal {P}}_t) \psi ({\mathcal {P}}_t)\frac{1}{c{{\widetilde{{\mathcal {R}}}}}_t} }\,dB^-_t, \\ d{{\widetilde{{\mathcal {R}}}}}_t&={{\widetilde{{\mathcal {R}}}}}_t\Big \{ \beta _0 {\mathcal {P}}_t\Big (1-\frac{c}{c_0}{{\widetilde{{\mathcal {R}}}}}_t\Big ) +\beta _1(1-{\mathcal {P}}_t)\Big (1-\frac{c}{c_1}{{\widetilde{{\mathcal {R}}}}}_t\Big )\Big \}\,dt\\&\quad +\sqrt{\frac{{{\widetilde{{\mathcal {R}}}}}_t}{c} \psi (1-{\mathcal {P}}_t)}\,dB^+_t. \end{aligned}$$

A special case of interest is obtained by taking $\beta _0/c_0=\beta _1/c_1=\alpha /c$, $\tau _0=\tau _1=\tau$, using a new parameter $\alpha$. For simplicity, let us also take $\rho _{01}=\rho _{10}=0$. Then

$$\begin{aligned} d{\mathcal {P}}_t&=(\beta _0-\beta _1){\mathcal {P}}_t(1-{\mathcal {P}}_t)\,dt + \sqrt{ {\mathcal {P}}_t(1-{\mathcal {P}}_t)\frac{\tau }{c{{\widetilde{{\mathcal {R}}}}}_t} }\,dB^-_t, \\ d{{\widetilde{{\mathcal {R}}}}}_t&={{\widetilde{{\mathcal {R}}}}}_t\Big \{ \beta _0{\mathcal {P}}_t +\beta _1(1-{\mathcal {P}}_t) -\alpha {\mathcal {R}}_t \Big \}\,dt +\sqrt{\tau {{\widetilde{{\mathcal {R}}}}}_t/c} \,dB^+_t. \end{aligned}$$

In this example we notice that the growth of species richness is diversity- and density-dependent while the selection term is now density-independent.

Let us now come back to the general model studied in this section and suppose that as $c\rightarrow \infty$ then both of $c_0$ and $c_1$ also tend to infinity, such that $c/c_i\rightarrow \alpha _i$, $i=0,1$, for some positive constants $\alpha _0$ and $\alpha _1$. Then formally

16 $$\begin{aligned} \begin{aligned} p_t'&=p_t(1-p_t) \big (\beta _0(1-\alpha _0\tilde{r}_t)-\beta _1(1-\alpha _1{{\tilde{r}}}_t)\big ) -\rho _{01}p_t +\rho _{10}(1-p_t), \\ {{\tilde{r}}}_t'&={{\tilde{r}}}_t \big (\beta _0 p_t(1-\alpha _0{{\tilde{r}}}_t) +\beta _1(1-p_t)(1-\alpha _1{{\tilde{r}}}_t)\big ). \end{aligned} \end{aligned}$$

The case $\beta _0=\beta _1=\beta >0$ gives the system of ODEs

$$\begin{aligned} p_t'&=\beta (\alpha _1-\alpha _0)\,p_t(1-p_t) {{\tilde{r}}}_t -\rho _{01}p_t +\rho _{10}(1-p_t), \\ {{\tilde{r}}}_t'&=\beta {{\tilde{r}}}_t \big (1-\tilde{r}_t(\alpha _0p_t+\alpha _1(1-p_t))\big ). \end{aligned}$$

The equation for *p* above shows that selection, positive or negative, strengthens with increasing species richness. In ecology, this would be equivalent to *K*-selection, whereby the trait less sensitive to competition is favored. For example, if $c_0>c_1$ so that trait 0 is less constrained than trait 1 then the selective rate of growth of trait 0 is positive, $\beta (\alpha _1-\alpha _0)>0$. For both “r-like” and “K-like” cases, selection is diversity-dependent. Returning to (16) and the special case $\alpha _0 \beta _0 = \alpha _1 \beta _1=\alpha$, although total species richness is regulated, species selection becomes diversity-independent and the selection coefficient reduces to $\beta _0 - \beta _1$. This would correspond to a scenario where the total diversity is regulated from mechanisms unrelated to the focal traits.

In macroevolution, several traits have been supposed to be evolutionary dead-ends, such as asexuality (Maynard-Smith 1978) or self-fertilization (Igic and Busch 2013). The classical view is that negative diversification is associated with such traits but they are continuously reintroduced through asymmetrical (or even unidirectional) shifts from traits associated with positive diversification (Goldberg et al. 2010). The above formulation suggests a more elaborate scenario whereby the dead-end trait has not necessarily a basal negative diversification rate but is more sensitive to diversity dependence. Such traits could lead to initial diversification in initially species-poor environments (i.e. much lower than the species carrying capacity) but diversification would then decrease and eventually be negative through time, as the number of species would increase. This is in agreement with recent empirical observations in the plant genus *Capsella* that selfing species are more sensitive to competition than outcrossing ones (Petrone Mendoza et al. 2018; Yang et al. 2018), and a plausible explanation for the hypothesis that selfing and asexual lineages “senesce” in diversification rates (Ho and Agrawal 2017).

### Fixation and extinction of traits

To study trait fixation probabilities using a similar approach as in Parsons et al. (2008) we let $\rho _{ 01}=\rho _{10}=0$ in Eq. (15), which means that species are unable to change traits. The initial composition of traits in the scaled species family at time 0 is a fraction *x* of trait 0 and the remaining fraction $1-x$ consisting of trait 1 species, $0<x<1$. As in Parsons et al. (2008), we strive to analyze the fate of the trait 0 element over time as a function of *x*. For this aim we must restrict further our considerations to the density dependent case with symmetric net growth rates, $\beta _0=\beta _1=\beta$, and a single carrying capacity *c*, the same for both traits. Then

17 $$\begin{aligned} d{\mathcal {P}}_t&=-{\mathcal {P}}_t(1-{\mathcal {P}}_t)(\tau _0-\tau _1)\frac{1}{{\mathcal {R}}_t}\,dt + \sqrt{ {\mathcal {P}}_t(1-{\mathcal {P}}_t) \psi ({\mathcal {P}}_t) \frac{1}{{\mathcal {R}}_t}}\,dB^-_t,\nonumber \\ d{\mathcal {R}}_t&=\beta {\mathcal {R}}_t \Big (1-\frac{{\mathcal {R}}_t}{c}\Big )\,dt +\sqrt{{\mathcal {R}}_t \psi (1-{\mathcal {P}}_t)}\,dB^+_t, \end{aligned}$$

which is a logistic version of the model listed as special case (a) in Sect. 3.6. The crucial aspect of the resulting SDE for ${\mathcal {P}}$, which is required for our method of proof to work, is that all dependence on ${\mathcal {R}}$ is mediated through a single function $g({\mathcal {R}})$, in this case $g(r)=1/r$, which appears as a multiple in both the diffusion drift term and the diffusion variance term. This is the reason why the closely related special case $\beta _0=\beta _1$, $c_0\not = c_1$ is not covered by our method, nor is $\beta _0\not =\beta _1$, $c_0=c_1$ or $\tau _0=\tau _1$, $\beta _0\not =\beta _1$.

In the present situation, however, general properties of logistic branching processes show that the species richness ${\mathcal {R}}$ goes extinct with probability one as $t\rightarrow \infty$. We may then consider the sequence of stopped processes $({\mathcal {P}}_{t\wedge \delta _n},{\mathcal {R}}_{t\wedge \delta _n})_{t\ge 0}$, where $\delta _n=\inf \{t>0: {\mathcal {R}}_t=1/n\}$, and have a well-defined solution of (17) with positive richness component on $\{0\le t\le \delta _n\}$ for each fixed *n*. Yet, the mixture of traits as captured by $({\mathcal {P}}_{t\wedge \delta _n})_{t\ge 0}$ along the path to extinction of the species family, as $n\rightarrow \infty$, would perhaps be of limited interest, indicating that the fixation probability concept might have limited relevance. On the other hand, the quasi-stationary behavior of ${\mathcal {R}}$ mentioned previously means that the time to extinction can be very long. This allows us to circumvent species extinction by modeling the species family rather using a process ${\mathcal {R}}^+$, say, which is ${\mathcal {R}}$ conditioned on the event $\{{\mathcal {R}}_t>0,t>0\}$ of ultimate nonextinction. In the following we study trait absorption with respect to such non-extinct paths. Let $({\mathcal {P}}^+,{\mathcal {R}}^+)$ denote the solution of (17) conditioned on the event of ultimate nonextinction. The boundaries 0 and 1 are both absorbing for ${\mathcal {P}}^+$. Let us denote by $\eta _0$ the fixation time of trait 1, that is, the random time at which ${\mathcal {P}}^+$ first hits the lower boundary 0, if ever. Similarly, $\eta _1$ is the fixation time of trait 0, the time at which the upper boundary point 1 is first hit. If $\eta _0$ is finite, then all species are trait 1 from that time and onward, meaning that the upper limit is never reached, $\eta _1=\infty$. Similarly, if $\eta _1<\infty$ all species end up as trait 0, and $\eta _0=\infty$. The absorption time $\eta =\min (\eta _0,\eta _1)$ is the time of extinction or fixation, whichever occurs first. Next we recover the fixation probability (5) within our setting, and derive a result on the “species frequency spectrum”.

#### Theorem 1

The fixation probability $q(x)={\mathbb {P}}_x(\eta _1<\infty )$ of ${\mathcal {P}}^+$ rendering all species trait 0 eventually, given initial frequency *x* of trait 0, equals

$$\begin{aligned} q(x)=\frac{\tau _1x}{\tau _0(1-x)+\tau _1x}. \end{aligned}$$

Moreover, for bounded functions *f* defined on the unit interval [0, 1],

$$\begin{aligned} {\mathbb {E}}_x\Big [\int _0^{\eta ^+} \frac{f({\mathcal {P}}^+_s)}{{\mathcal {R}}_s^+}\,ds\Big ]= \frac{2}{\tau _0(1-x)+\tau _1x} \Big \{ x\int _x^1\frac{f(y)}{y}\,dy+(1-x)\int _0^x\frac{f(y)}{1-y}\,dy\Big \}. \end{aligned}$$

#### Proof

Let $X=(X_t)_{t\ge 0}$ be a diffusion process, which solves

18 $$\begin{aligned} dX_t=-X_t(1-X_t)(\tau _0-\tau _1)\,dt + \sqrt{X_t(1-X_t) \psi (X_t)}\,dB^-_t. \end{aligned}$$

The SDE for *X* is the same as that of ${\mathcal {P}}^+$ under the enforced condition ${\mathcal {R}}^+\equiv 1$. It turns out that the fixation probabilities of ${\mathcal {P}}^+$ and *X* respectively, coincide. The reason for this is that the distribution of *X* can be extracted from that of ${\mathcal {P}}^+$, via a random time change. Since ${\mathcal {R}}^+_t>0$ for all *t*, the transform of ${\mathcal {R}}^+$, given by the function

$$\begin{aligned} B_t= \int _0^t \frac{1}{{\mathcal {R}}_s^+}\,ds,\quad t>0, \end{aligned}$$

is a strictly increasing random time change with time change rate $1/{\mathcal {R}}_t^+$, such that its left-inverse

$$\begin{aligned} A_t=\inf \{s>0: B_s>t\}, \quad t>0, \end{aligned}$$

is continuous, and $A_{B_t}=t$ for all $t\ge 0$. Thus,

$$\begin{aligned} q(x)={\mathbb {P}}_x(\eta _1^+<\infty )={\mathbb {P}}_x(B_{\eta ^+}<\infty ),\quad B_{\eta ^+}=\inf \{t>0: {\mathcal {P}}^+_{A_t}=1\}. \end{aligned}$$

The desired function *q*(*x*) is now obtained as the fixation probability of the time-changed process $({\mathcal {P}}^+_{A_t})_{t\ge 0}$, which turns out to be closely connected to *X*. Indeed, it is a consequence of the time change result Theorem 8.5.1 in Øksendal (2007), that, for each $t\ge 0$, ${\mathcal {P}}_{A_t}^+$ and $X_t$ have the same distribution. Let $\eta _1^X$ denote the fixation time of *X*. Since

$$\begin{aligned} {\mathbb {P}}_x(\eta _1^X>t)={\mathbb {P}}_x(X_t=1)={\mathbb {P}}({\mathcal {P}}_{A_t}^+=1) = {\mathbb {P}}_x(B_{\eta ^+}>t), \end{aligned}$$

it follows that $\eta _1^X$ and $B_{\eta _1^+}$ have the same distribution, and so $q(x)={\mathbb {P}}(\eta _1^X<\infty )$. Similarly, $\eta ^X$ and $B_{\eta ^+}$ have the same distribution.

The SDE for *X* in Eq. (18) is

$$\begin{aligned} dX_t=b(X_t)\,dt +\sigma (X_t)\,dB^-_t,\quad b(x)=(\tau _1-\tau _0)x(1-x),\quad \sigma ^2(x)=x(1-x)\psi (x), \end{aligned}$$

with $\psi (x)=\tau _0(1-x)+\tau _1x$ introduced in Eq. (14), and where $X_0=x$, $0<x<1$, and the boundary points $\{0,1\}$ are absorbing. The scale function *S*(*x*) and speed function *m*(*x*) associated with $(X_t)_{t\ge 0}$, $X_0=x$, are defined by

$$\begin{aligned} S(x)=\int _0^x s(y)\,dy,\quad m(x)=\frac{1}{\sigma ^2(x)s(x)},\quad 0<x<1, \end{aligned}$$

where

$$\begin{aligned} s(x)=\exp \Big \{-\int _0^x\frac{2b(y)}{\sigma ^2(y)}\,dy\Big \}, \end{aligned}$$

Here,

$$\begin{aligned} S(x)=\frac{\tau _0x}{\tau _0(1-x)+\tau _1 x},\quad m(x)=\frac{\tau _0(1-x)+\tau _1x}{\tau _0^2x(1-x)}. \end{aligned}$$

By using Feller’s boundary classification and the theory of one-dimensional diffusion processes (Karlin and Taylor 1981; Etheridge 2011), it follows that the boundary points 0 and 1 are both accessible from the interior state space as exit boundaries. The extinction probability is determined by the scale function (using the normalization $S(0)=0$), as

$$\begin{aligned} q(x)=\frac{S(x)}{S(1)}=\frac{\tau _1x}{\tau _0(1-x)+\tau _1 x}. \end{aligned}$$

Moreover, for bounded functions *f*,

$$\begin{aligned} {\mathbb {E}}_x\Big [\int _0^{\eta ^X}f(X_s)\,ds\Big ]&=2q(x)\int _x^1 (S(1)-S(y))m(y)f(y)\,dy\\&\quad +2(1-q(x)) \int _0^x S(y)m(y)f(y)\,dy. \end{aligned}$$

It is straightforward to check that the right hand side equals

$$\begin{aligned} \frac{2}{\tau _0(1-x)+\tau _1x} \Big \{ x\int _x^1\frac{f(y)}{y}\,dy+(1-x)\int _0^x\frac{f(y)}{1-y}\,dy\Big \}. \end{aligned}$$

To complete the proof, we first use once again that ${\mathcal {P}}^+_{A_t}$ and $X_t$ have the same distribution for each fixed *t*, and then make the change-of-variables $s=B_r$, to obtain

$$\begin{aligned} {\mathbb {E}}_x\Big [\int _0^{\eta ^X} f(X_s)\,ds\Big ]&= {\mathbb {E}}_x\Big [\int _0^\infty 1_{\{0<X_s<1\}}f(X_s)\,ds\Big ]\\&= {\mathbb {E}}_x\Big [\int _0^\infty 1_{\{0<{\mathcal {P}}^+_{A_s}<1 \}}f({\mathcal {P}}^+_{A_s})\,ds\Big ]\\&= {\mathbb {E}}_x\Big [\int _0^{B_{\eta ^+}}f({\mathcal {P}}^+_{A_s})\,ds\Big ] = {\mathbb {E}}_x\Big [\int _0^{\eta ^+}\frac{f({\mathcal {P}}^+_r)}{{\mathcal {R}}^+_r}\,dr\Big ]. \end{aligned}$$

$$\square $$

### Application of Theorem 1: fixation and extinction of a rare trait

Suppose we have a family in which all species carry a single trait, and we introduce a species with a second trait of the same net growth rate into the population. What are the chances of the new trait getting fixed or lost? What are the implications if the new trait causes a shift in turnover rate? For example, all else being equal, this would correspond to a shift in life span, lifespan being inversely proportional with respect to turnover rate. As another example, recently, polyploidy (the doubling of the genome) has been shown to have no effect on diversification ($\beta _0 = \beta _1$) but to increase turnover rate ($\tau _0 > \tau _1$) (Zenil-Ferguson et al. 2019).

To address these questions we observe that if a small fraction *x* of trait-0 species are inserted into a population otherwise consisting entirely of trait-1 species, then as $x\rightarrow 0$,

$$\begin{aligned} q(x)=\frac{\tau _1 x}{\tau _0(1-x)+\tau _0x} \sim \frac{\tau _1}{\tau _0}x. \end{aligned}$$

In addition, by an application of the second statement in Theorem 1 with $f(y)=1$, $y\in [0,1]$, the expected absorption time weighted by the inverse species richness process, satisfies

$$\begin{aligned} {\mathbb {E}}_x\Big [\int _0^{\eta ^+} \frac{1}{{\mathcal {R}}_s^+}\,ds\Big ]= \frac{-2x\ln (x)-2(1-x)\ln (1-x)}{\tau _0(1-x)+\tau _1x} \sim \frac{-2x\ln (x)+2x}{\tau _0}, \end{aligned}$$

as $x\rightarrow 0$. Keeping the turnover rate $\tau _1$ of the original trait fixed, we see that the probability of fixation of the new trait is inversely proportional to the turnover rate $\tau _0$ of the new trait. Thus, traits which cause a burst in both speciation and extinction are suppressed whereas traits with low turnover rates are favored, with respect to possible fixation. Similarly, the expected weighted absorption time decreases with increasing turnover rate $\tau _0$, consistent with shorter time to extinction.

An alternative interpretation of the result in Theorem 1 suggests a notion of trait frequency spectrum in analogy to the allele frequency spectrum in population genetics. For this we assume, with reference to the space and time scaling parameter *n*, that in each of the *n* time steps forming one evolutionary time unit, trait injections occur at rate $\theta >0$. When one of these events happens, a fraction 1/*n* of the family gets a new trait of turnover rate $\tau _0$. The remaining fraction has turnover rate $\tau _1$. We think of the successive injection events representing each time a new trait unrelated to previous ones. Each new trait traces out its own path ${\mathcal {P}}^+$ but all relate to the same ${\mathcal {R}}^+$. Most of the new traits quickly go extinct. A few might survive for a while and even get fixed, eventually. The scaled trait 0 fixation probability per time unit is the large *n* limit

$$\begin{aligned} nq(1/n)\rightarrow \frac{\tau _1}{\tau _0}. \end{aligned}$$

A possible interpretation is that with larger turnover rate $\tau _0$ of an “invading” trait, the smaller is the fixation rate $\tau _1/\tau _0$, and hence, the more efficient is the existing species family in purging such an intruder. For non-negative bounded functions *f* on the interval [0, 1] with $f(0)=0$, which satisfies the integrability condition $\int _0^1 f(y)y^{-1}\,dy<\infty$, we find

$$\begin{aligned} \lim _{n\rightarrow \infty } \theta n {\mathbb {E}}_{1/n}\Big [\int _0^{\eta ^+}\frac{f({\mathcal {P}}^+_r)}{{\mathcal {R}}^+_r}\,dr\Big ] = \frac{\theta \tau _1}{\tau _0} \int _0^1f(y) \frac{2}{\tau _1y}\,dy = \int _0^1f(y) \frac{2\theta }{\tau _0y}\,dy. \end{aligned}$$

In particular,

$$\begin{aligned} \lim _{n\rightarrow \infty } \theta n {\mathbb {E}}_{1/n}\Big [\int _0^{\eta ^+}\frac{{\mathcal {P}}^+_r}{{\mathcal {R}}^+_r}\,dr\Big ] = \frac{2\theta }{\tau _0}. \end{aligned}$$

The weight function $2\theta \tau _0^{-1}/y$ which arises in the above scheme of limiting expected values, plays a similar role as the stationary allele frequency spectrum in population genetics. While such a frequency weight function is not integrable over $y\in (0,1)$, and hence does not allow a probability density interpretation, it does define an intensity measure for a well-defined Poisson random measure on (0, 1). For the Poisson random field approach in population genetics, see Sawyer and Hartl (1992) and e.g. Kaj and Mugal (2016), Section 2.3. Intuitively, for each $y\in (0,1)$, $(2\theta /\tau _0) y^{-1}$ is in this sense the stationary intensity that $({\mathcal {P}}_t)_{t\ge 0}$ occupies the frequency band near *y*, as measured by the size of $({\mathcal {P}}_t^+/{\mathcal {R}}_t^+)_{t\ge 0}$ within the time frame during which the trait remains in the family.

The case $\tau _0=\tau _1$ is the fully neutral case with fixation probability $q(x)=x$ and trait spectrum intensity $2\theta /y$. The case $\tau _0\not =\tau _1$ represents a form of trait selection, which only affects the relative magnitude of trait frequencies present in the species family, but not the shape of the trait frequency spectrum.

## The super-process representation of the limit process

In this final section, we return to the two-type branching process *X* with rates (1) running on the scale of species generations. The aim is to provide a sketch of the proof of the limit result Proposition 1, shifting to the evolutionary time scale.

As an alternative and from a wider perspective, we may consider *X* as a measure-branching process with spatial motion on the trait space $E=\{0,1\}$ and spatially dependent binary branching. Each lineage in the branching tree changes its current species trait according to independent copies of the space motion $J=(J_u)$, which is the two-state Markov jump process with jump rates $\delta _{01}$ and $\delta _{10}$ for transitions from 0 to 1 and vice versa. In addition to the on/off motion caused by *J*, the number of species of each trait develop independently as linear binary branching processes with generating functions

$$\begin{aligned} F(z;j)=\frac{\lambda _j z^2+\mu _j}{\lambda _j+\mu _j} =\frac{d_j+\tau _j}{2\tau _j}z+\frac{\tau _j-d_j}{2\tau _j} ,\quad j=0,1. \end{aligned}$$

The branching offspring distribution for daughter species of trait *j* has mean $d_j/\tau _j$, and variance $(\tau _j-d_j)/\tau _j$. Now let us put $\langle X_u,f\rangle = f(0)K_u+f(1)L_u$, $u\ge 0$, where $f=(f(0),f(1))$ is a function (row vector) on *E*. We write ${\mathbb {E}}_j$ for the expected value conditional on $J_0=j$, that is $X_0=(1,0)$ when $j=0$ and $X_0=(0,1)$ when $j=1$. Then *X* is the measure-branching process for which the function $v_u$ defined by

$$\begin{aligned} v_u(j)={\mathbb {E}}_j[e^{\langle X_u,f\rangle }-1], \end{aligned}$$

is the unique solution of the integral equation

19 $$\begin{aligned} v_u(j)={\mathbb {E}}_j[e^{f(J_u)}-1]+{\mathbb {E}}_j \int _0^u \big (F(1+v_{u-r}(J_r);J_r)- 1-v_{u-r}(J_r)\big ) \tau _{J_r}\,dr, \end{aligned}$$

for $j\in E$, cf. Li (2011), Ch. 4.1. It is well-known that suitably scaled measure-valued branching processes converge in the limit of many particles, long times, and small masses to an associated superprocess, in this case a super-on/off process. More exactly, the superprocess is the weak limit as *n* independent copies $X^{(k,n)}$, $k=1,\dots ,n$, of *X* with spatial motion $J^{(n)}$ and branching mechanism $F^{(n)}(z,;)$ scaled according to (6), are averaged over the time span $u=nt$. This, however, is the species model under evolutionary time scaling, and hence the superprocess will be the limit ${\mathcal {X}}$ of ${\mathcal {X}}_t^{(n)}=n^{-1}X_{nt}^{(n)}$, $t\ge 0$, in the sense of weak convergence in path space as $n\rightarrow \infty$. To study the limit process we put

$$\begin{aligned} V_t^{(n)}[f](j)=n{\mathbb {E}}_j[e^{ \langle X_{nt}^{(n)},f/n\rangle }-1], \end{aligned}$$

so that $V_t^{(1)}[f](j)=v_t(j)$. Then, for large *n*,

$$\begin{aligned} \ln {\mathbb {E}}_j[ e^{\langle {\mathcal {X}}_t^{(n)},f\rangle }]&= n \ln {\mathbb {E}}_j\Big [ \exp \{ \langle X_{nt}^{(n)},f/n\rangle \}\Big ] \\&= n\ln \Big (1+\frac{n{\mathbb {E}}_j[ e^{ \langle X_{nt}^{(n)},f/n\rangle }-1]}{n}\Big ) \sim V^{(n)}_t[f](j). \end{aligned}$$

By using once more the scaling assumption (6), we have

$$\begin{aligned} P(J^{(n)}_{n(t+h)}=1|J^{(n)}_{nt}=0)=\delta _{01}^{(n)}nh +\frac{o(nh)}{n}= \rho _{01}h+o(h), \end{aligned}$$

and the analogous relation for converse transitions from 1 to 0. This means that the limit process of $J^{(n)}_{nt}$, $t\ge 0$, is again an on/off process on *E* but now with jump rates $\rho _{01}$, $\rho _{10}$. For simplicity we retain the notation *J* in the limit. Also, a calculation shows,

$$\begin{aligned} n^2\big (F^{(n)}(1+v/n;j)-1-v/n\big )\tau _j^{(n)}&= \frac{n^2}{2}\Big (\! \tau _j^{(n)}(v/n)^2+d_j^{(n)}(2(v/n)+(v/n)^2)\! \Big )\\&\sim \beta _j v+\tau _j \frac{v^2}{2}. \end{aligned}$$

As we combine these observations with Eq. (19), it follows that the function $V^{(n)}[f](j)$ satisfies

$$\begin{aligned} V^{(n)}_t[f](j)&={\mathbb {E}}_j[n(e^{f(J_t)/n}-1)]\\&\quad +{\mathbb {E}}_j\int _0^t \big ( \beta _{J_r} V^{(n)}_{t-r}[f](J_r) + \tau _{J_r} V^{(n)}_{t-r}[f](J_r)^2/2\big ) \,dr+H_t^{(n)}, \end{aligned}$$

where the remainder term $H^{(n)}$ can be controlled and shown to vanish in the limit $n\rightarrow \infty$, see Ch. 4.2 in Li (2011). With reference to the theory of superprocesses we can now conclude that ${\mathcal {X}}^{(n)}$ possesses a weak limit process ${\mathcal {X}}$, such that $V_t[f](j)=\ln {\mathbb {E}}_j[ e^{\langle {\mathcal {X}}_t,f\rangle }]$ is the unique solution of the integral equation

$$\begin{aligned} V_t[f](j)={\mathbb {E}}_j[f(J_t)] +\int _0^t {\mathbb {E}}_j \big [ \beta _{J_r} V_{t-r}[f](J_r) + \tau _{J_r} V_{t-r}[f](J_r)^2/2\big ] \,dr,\quad j\in E, \end{aligned}$$

cf. Prop. 4.5 in Li (2011). The associated stochastic equation for the superprocess is

20 $$\begin{aligned} {\langle {\mathcal {X}}_t,f\rangle }={\langle {\mathcal {X}}_0,f\rangle }+\int _0^t {\langle {\mathcal {X}}_s,{\mathcal {G}}f\rangle }\,ds +\int _0^t {\langle {\mathcal {X}}_s,\beta f\rangle }\,ds+M_t(f). \end{aligned}$$

Here, ${\mathcal {G}}f$ is the infinitesimal generator of *J*, defined by

$$\begin{aligned} {\mathcal {G}}f(0)=(f(1)-f(0))\rho _{01},\quad {\mathcal {G}}f(1)=(f(0)-f(1))\rho _{10}, \end{aligned}$$

and *M*(*f*) is a continuous martingale with representation

$$\begin{aligned} M_t(f)=f_0 \int _0^t \sqrt{\tau _0 {\mathcal {K}}_s}\,dB_s^0+ f_1 \int _0^t \sqrt{\tau _1 {\mathcal {L}}_s}\,dB_s^1, \end{aligned}$$

using a 2-dimensional standard Brownian motion $(B^0,B^1)$ with independent components. Hence the quadratic variation of *M*(*f*) is

$$\begin{aligned} \langle \!\langle M(f),M(f)\rangle \!\rangle _t = \int _0^t \langle {\mathcal {X}}_s, \tau f^2\rangle \,ds. \end{aligned}$$

By observing that $\langle {\mathcal {X}},f\rangle _t$ is the scalar product $f\cdot {\mathcal {X}}_t$ of the vectors *f* and ${\mathcal {X}}_t$ and that

$$\begin{aligned} \langle {\mathcal {X}}_s,{\mathcal {G}}f\rangle + \langle {\mathcal {X}}_s,\beta f\rangle = f\cdot A {\mathcal {X}}_s, \end{aligned}$$

it follows that the equation for ${\mathcal {X}}$ in Proposition 1 is an alternative representation of Eq. (20), cf. Li (2011), Ch. 7.5.

Conditional on ultimate survival, the results for $({\mathcal {P}},{\mathcal {R}})$ now follow from Ito’s formula with

$$\begin{aligned} B^+_t=\int _0^t \sqrt{\frac{\tau _0P_s}{\tau _0P_s+\tau _1(1-P_s)}}\,dB^0_s +\int _0^t \sqrt{\frac{\tau _1(1-P_s)}{\tau _0P_s+\tau _1(1-P_s)}}\,dB^1_s, \end{aligned}$$

and

$$\begin{aligned} B^-_t =\int _0^t \sqrt{\frac{\tau _0(1-P_s)}{\tau _0(1-P_s)+\tau _1 P_s}}\,dB^0_s - \int _0^t \sqrt{\frac{\tau _1 P_s}{\tau _0(1-P_s)+\tau _1 P_s}}\,dB^1_s. \end{aligned}$$

## Conclusions

In this work, we presented a stochastic modeling framework for a binary trait-dependent species family. The model provides insights on the joint evolution of trait frequencies and species diversity. Guided by the long-term successful use of stochastic techniques in population genetics, we applied various probabilistic methods to study the effects of diversity-dependence and related properties in the species family, hence developing a phylogenetic methodology. At the core of the study is the interpretation that phylogenetic trait frequency and population genetics allele frequency are closely related from the view point of stochastic models. We showed that evolutionary time re-scaling is a powerful tool to reveal relevant analogies and distinctions, and to provide a bridge between two seemingly distant application areas.

We applied methods, which rely on the analysis of trajectories of solutions, to stochastic differential equations of Wright–Fisher type with diversity dependent selection and genetic drift coefficients. Our main structural result, Proposition 1, derived from an embedding argument into an abstract two-type superprocess, provides the precise dynamics of trait frequency jointly with species richness. In parallel, we also discussed the corresponding Markov generator dynamics, referred to as the Gillespie approach. In doing so, we were able to resolve a paradoxical conclusion in Gillespie (1974), dealing with the effective population size. To put these abstract results into context, we discussed a number of special cases with partly symmetric parameter settings leading up to a fully symmetric, or neutral, case. To help clarify the role of parameters, we identified trait success (or trait fitness) functions, which are further utilized for the application of the model to diversity-dependent interaction in terms of carrying capacity. A particular logistic version of the diversification-dependent model was the subject of an in-depth study of fixation and extinction of traits. The main result, Theorem 1, provides the fixation probability as function of the initial frequency of a newly injected trait, as well as a type of trait frequency spectrum, which is analogous to allele frequency spectrum in population genetics.
